# Are Diminishing Potentiation and Large Extensor Moments the Cause for the Occurrence of the Sticking Region in Maximum Free-Weight Barbell Back Squats among Resistance-Trained Males?

**DOI:** 10.5114/jhk/185720

**Published:** 2024-04-15

**Authors:** Roland van den Tillaar, Hallvard Nygaard Falch, Stian Larsen

**Affiliations:** 1 Department of Sports Sciences and Physical Education, Nord University, Levanger, Norway.

**Keywords:** resistance exercise, sticking point, strength, kinetics, kinematics

## Abstract

This study compared the kinematics, surface electromyography (sEMG) and kinetics among isometric squats performed at 10 different heights of the upward part and a one-repetition maximum (1-RM) squat. Eleven males (age: 27.5 ± 3.4 years, body mass: 84.9 ± 8.1 kg, body height: 1.79 ± 0.06 m, 1-RM squat: 152.2 ± 20.55 kg) took part in this study. It was found that force output was lowest in the sticking region at around the event of peak deceleration for the 1-RM trial with force output at 2179 ± 212 N. For the isometric trial, the lowest force output occurred at the lowest barbell height (1735 ± 299 N). In addition, for the 1-RM condition hip extension moments peaked at the first four barbell heights (6.5–6.2 Nm/kg) representing the pre-sticking and the sticking region before significantly decreasing during the events representing the post-sticking region. Additionally, the sEMG amplitude peaked for the hip extensors at the barbell heights corresponding to the post-sticking region. Moreover, the sEMG amplitude was significantly higher for the 1-RM condition for all hip extensors, vastus lateralis, and calf muscles (F ≥ 2.7, p ≤ 0.01, η_p_^2^ ≥ 0.25). Therefore, we suggest that the sticking region occurs because of reduced force output in the pre-sticking and the sticking region in back squats among resistance-trained males. The reduced force output is probably a combination of suboptimal internal moment arms, length-tension relationships of the gluteus maximus, hamstring and vastii muscles in the pre-sticking and sticking regions to overcome the large extensor moments together with diminishing potentiation from the pre-sticking to the sticking region.

## Introduction

The back squat is a popular resistance exercise to develop maximal strength in the lower extremities (Schoenfeld, 2010). A successful back squat is typically performed by flexing the hip and knee joints to the hips lower than the knees before ascending back to the start position (Larsen et al., 2021a). At maximal and submaximal percentages of 1-repetition maximum (1-RM), a sticking region occurs during the upward phase (Kompf and Arandjelović, 2017). It has been proposed that the hip extensors may have a disadvantageous length-tension relationship, resulting in incapability to produce maximal force due to being lengthened past the plateau of the length-tension relationship during the lower barbell heights of a full squat (Escamilla et al., 2001; Larsen et al., 2021b; Spieszny et al., 2022). In addition to the muscle mechanics, it has been proposed that barbell deceleration occurs in the sticking region because the increased force output during ascent is reduced due to diminishing muscle potentiation (van den Tillaar and Ettema, 2010).

Therefore, to test this hypothesis, van den Tillaar et al. (2021) compared kinematics, kinetics, and the sEMG amplitude among a 1-RM Smith-machine full squat and isometric squats performed at 10 different barbell heights from the lowest barbell height (deepest point during the whole lift) to the end of the upward phase (standing fully upright). Those authors hypothesized that force output would be lower in the sticking region, supporting that this is a poor biomechanical position. In addition, they hypothesized that the sEMG amplitude would be higher under the 1-RM condition, indicating that potentiation due to re-use of stored elastic energy, more active muscles and/or the stretch reflex occurs (Bobbert et al., 1996; Walshe et al., 1998). Findings from that study were that force output was lowest between 0 and 15 cm from the lowest barbell height, supporting their first hypothesis. Moreover, a higher sEMG amplitude was found for the 1-RM condition for almost all muscles when compared with the isometric squat condition, which they suggested supported their second hypothesis that muscle potentiation may occur (van den Tillaar et al., 2021). However, the authors pointed out two major limitations of their study. Firstly, the ankle dorsal flexion angle during the upward phase was not controlled, which may have affected the data. Secondly, no inverse dynamics analysis was conducted due to insufficient equipment, which may give more information about the net joint moments at different heights (van den Tillaar et al., 2021). Moreover, maximal one-repetition maximum testing in the study was performed on a Smith machine, which may be different from free-weights because of fewer stability requirements (Cotterman et al., 2005).

Therefore, the intention of this study was to reproduce the study by van den Tillaar et al. (2021), but with controlled hip flexion, knee flexion, and ankle dorsal flexion angles performing isometric and 1-RM squats together with inverse dynamics analyses. Also, this study used free-weight barbell back squats during 1-RM testing and not the Smith machine as used in the study by van den Tillaar et al. (2021) to avoid force output forwards during the 1-RM trial and have a more ecological comparison with the free-weight barbell squat, which is a very popular exercise in strength training. We aimed to compare kinetics, barbell, and joint kinematics together with the sEMG amplitude of twelve different muscles at 10 different positions from the lowest barbell height, to cover a full range of motion during the upward phase. We hypothesized that hip and knee joint moments would peak together with low force output at the heights representing the sticking region, supporting that the sticking region occurs due to a poor mechanical position (Larsen et al., 2021b; van den Tillaar et al., 2020, 2021). In addition, we expected increased force output and a greater sEMG amplitude under the 1-RM condition compared to the isometric condition due to potentiation and/or better length-tension and force-velocity relationships (Hill, 1970) due to the descending phase under the 1-RM condition.

## Methods

### Participants

Eleven healthy males (body mass: 84.9 ± 8.1 kg, age: 27.5 ± 3.4 years, body height: 179.2 ± 6.4 cm) participated in this study, which has shown to be sufficient to identify difference in output force and sEMG based upon van den Tillaar et al. (2021). Inclusion criteria were: no injuries at the time of testing that could reduce maximal performance, and participants needed to be able to squat 1.5 x their own body mass. All participants were informed in writing and orally about study procedures and before participation a signed written consent form was gathered. The study was performed following the latest revision of the Declaration of Helsinki and current ethical regulations for research. The study was approved by the Norwegian Agency for Shared Services in Education and Research (approval code: 701688; approval date: 14 July 2000).

### Design and Procedures

To compare the kinematic, kinetic, and sEMG amplitude patterns in the upward phase among 1-RM squats and isometric squats at different barbell heights, a within-subject, repeated measures design was used. Ten different barbell heights with 6-cm differences between the heights from the lowest barbell height to the fully upright position were used. Additionally, barbell kinematics and kinetics were analyzed at the events’ lowest barbell height (v_0_), first maximal barbell velocity (v_max1_), first peak barbell deceleration (d_max1_), and first minimum barbell velocity (v_min_) also called the sticking point, and second peak barbell velocity (v_max2_) during the upward phase (Larsen et al., 2021a, 2021b). Dependent variables included mean barbell height, barbell velocity, time, hip, knee, and ankle joint flexion angles, vertical force, anteroposterior force, hip, knee, and ankle moment arms, hip, knee, and ankle extension moments, and the sEMG amplitude at ten different barbell heights.

Participants self-determined barbell placement, stance width of feet and external rotation, yet they were standardized for each participant through each condition. The depth requirement for v_0_ was standardized with depth requirements from the International Powerlifting Federation (IPF, 2019) and a horizontal attached band was used that needed to contact the hamstring before the participant was allowed to start the upward phase. Testing started with a warm-up, involving 3 sets of 6–10 repetitions with an Olympic barbell (Rogue, Ohio power bar). Thereafter one repetition at 70%, 90%, and 100% of 1-RM was performed. The load was decreased or increased by 2.5–5 kg until the real 1-RM was obtained. A four-min rest interval was given between each attempt to avoid fatigue and obtain maximal 1-RM performance (Rahimi, 2005). Participants performed the 1-RM with their own technique in their own tempo, down and upwards to avoid extra stress. A maximum of two-three attempts was necessary to obtain 1-RM for each participant. Participants performed maximal isometric squats in random order on a locked Smith machine 10 min after 1-RM testing (Powerline Smith Machine model: PSM144X, Body-Solid, Forest Park, IL, USA) with the same procedures as van den Tillaar et al. (2021). Maximal isometric squats were performed for around 3 s at 10 different heights with 6-cm differences between each height and in random order. This was to simulate, during the upward movement, the full range of motion. The rest interval between the different barbell heights was approximately 5 min to avoid fatigue. To compare the differences in the sEMG amplitude together with force output during 1-RM, for a 2-cm movement range around the same distances as the isometric conditions, average force and RMS of each muscle were calculated (van den Tillaar et al., 2021). In addition, for the isometric conditions, we used the highest force output over approximately 1 s, with the RMS sEMG amplitude during this time. This was compared with the 1-RM height. To standardize hip flexion, knee flexion, and ankle dorsal flexion angles among the 1-RM and isometric conditions, a pipeline in Visual 3D was used which automatically calculated joint angles at the different barbell heights. Participants were verbally instructed and guided by a research assistant to ensure similar joint kinematics for the isometric conditions as the 1-RM condition.

### Measures

**Figure 1:**
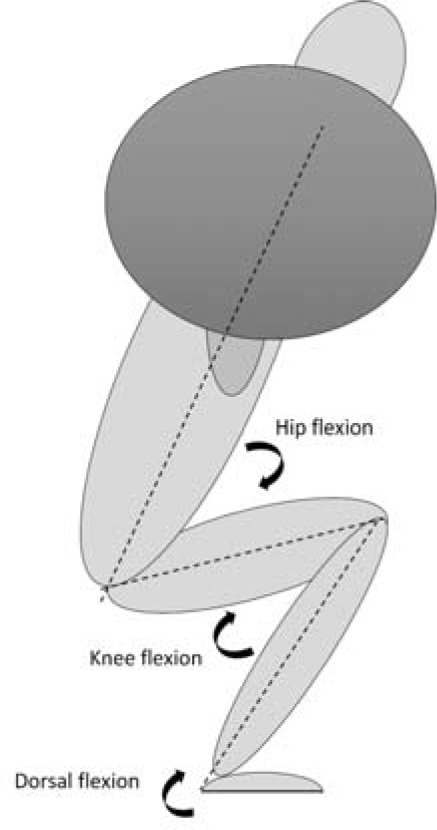
Definition of joint angles for the squat.

Trigno Avanti sensors (DELSYS inc, Natick, MA, USA) were used to record electromyography (EMG) activity on the participants' right leg for twelve different muscles: trapezius pars transversus, erector spinae longissimus, erector spinae iliocostalis, gluteus medius, gluteus maximus, vastus medialis, vastus lateralis, rectus femoris, biceps femoris, semitendinosus, soleus medialis, and gastrocnemius medialis with a sampling rate of 1111 Hz. Placement of electrodes was done according to the recommendations of SENIAM (Hermens et al., 2000). For reducing the impedance, the skin was shaved, rubbed with alcohol, and dried with paper before attaching sensors. EMG data were recorded and synchronized with body movements using a three-dimensional motion capture system with eight cameras at a sampling rate of 500 Hz (Qualisys, Gothenburg, Sweden), which were used to track reflective markers for motion capture data, and to determine joint angles of the hip, knee, and ankle joints in the sagittal plane. Hip and knee flexion was defined as 0°, while ankle dorsal flexion was defined as 90° in the fully upright position (Figure 1). The reflective markers were placed at anatomical landmarks on both sides of the body (1^st^ and 5^th^ proximal phalanx, tuber calcanei, medial and lateral malleolus, the medial and lateral condyle of the knee, trochanter major, posterior superior iliac spine, iliac crest, pelvis, sternum and acromion), creating a 3D measurement of participants. In addition, two reflective markers were placed on the lateral tips of the barbell in order to track barbell velocity. Barbell velocity was defined relative to the laboratory. Kinematic data were exported as C3D files to Visual 3D (C-motion, Germantown, USA) for segment building. Both EMG and joint kinematics were thereafter analyzed in Visual 3D v6 software. Here, a high-pass and low-pass (20 and 500 Hz) filter was used to filter the EMG signals. After that, EMG signals were rectified, and mean RMS was calculated. To track the 3D ground reaction forces and enable inverse dynamics calculation, two force plates (Kistler force plate, type 9260AA6, Winterthur, Switzerland) were integrated into the Qualisys motion capture system. The ground reaction force moment arms were calculated as the shortest anterior-posterior distance in the sagittal plane between the joint centers and the center of pressure. The joint moments calculated in this study were external net joint moments expressed as means and standard deviations with respect to the distal segments resolute coordinate systems. The net joint moments were summed between the left and right segments and normalized to the participants’ mass using default normalization. The net joint moments are expressed as Nm/kg.

### Statistical Analysis

To check for normality, we used the Shapiro-Wilk test. To compare potential differences in kinetics and the sEMG amplitude among the 1-RM and the different heights at the isometric attempts, a repeated 2 x (condition: 1-RM and isometric) x 10 (barbell height: 0–54 cm) analysis of variance for each of the variables was used. To compare joint angles, we used a one-way analysis of variance with repeated measures. Holm-Bonferroni post hoc analyses were performed to determine potential differences. All the results are presented as means ± standard deviations (SDs). If the assumption of sphericity was violated, we reported the Greenhouse-Geisser adjustments of the *p*-values. Effect sizes were evaluated with η_p_^2^ (partial eta squared), where < 0.01 to 0.06 constituted a small effect, < 0.06–0.14 constituted a medium effect, and > 0.14 constituted a large effect (Cohen, 1988). The *p*-value to reach significance was set at *p* < 0.05. Statistics were analyzed in SPSS version 27.0 (IBM Corp, Armonk, NY, USA).

## Results

The participants squatted 152.2 ± 20.55 kg successfully in the back squat. Detailed information about the barbell and joint kinematics in the events v_0_, v_max1_, d_max1_, v_min_, and v_max2_ is presented in Table 1.

**Table 1:** Barbell kinematics and joint kinematics during the different events in the back squat (mean ± SD).

Event	V_0_	V_max1_	D_max1_	V_min_	V_max2_
Barbell height (m)	0	0.05 ± 0.02	0.13 ± 0.05	0.21 ± 0.06	0.54 ± 0.06
Barbell velocity (m/s)	0	0.29 ± 0.07	0.23 ± 0.06	0.09 ± 0.05	0.81 ± 0.19
Time (s)	0	0.25 ± 0.07	0.46 ± 0.31	0.99 ± 0.45	2.03 ± 0.92
Hip flexion (°)	102.0 ± 8.8	101.0 ± 7.9	94.3 ± 8.2	87.4 ± 7.1	37.4 ± 13.1
Knee flexion (°)	123.1 ± 7.2	114.4 ± 6.9	97.4 ± 8.4	85.2 ± 6.2	45.4 ± 11.3
Ankle dorsal flexion (°)	103.4 ± 5.6	100.5 ± 4.7	95.1 ± 4.1	91.3 ± 3.2	85.4 ± 4.9

**Figure 2:**
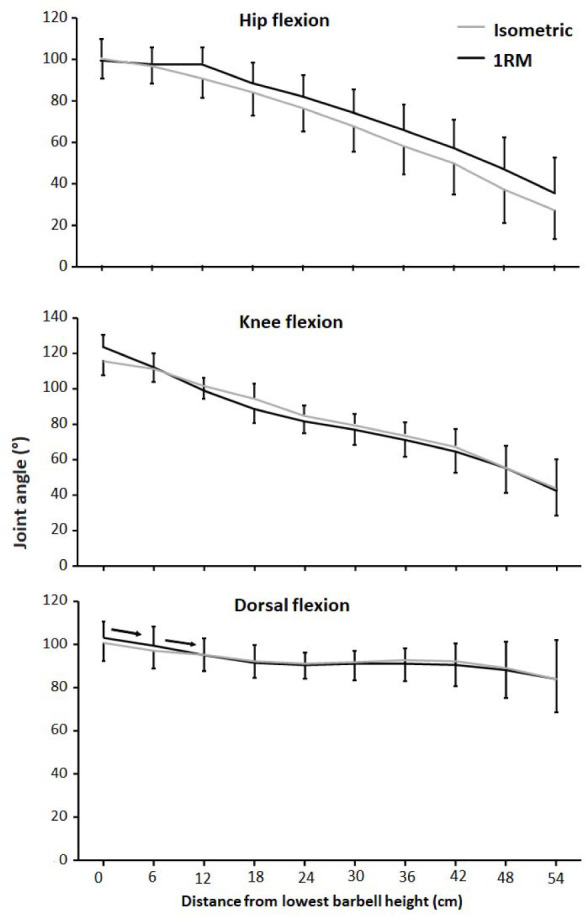
Mean (± SD) hip flexion, knee flexion, and ankle dorsal flexion angles at 10 vertical distances from v_0_ for the 1-RM and isometric conditions. → indicates a significant difference between these two barbell heights for both conditions at *p* ≤ 0.05

No differences were found between the two conditions for hip flexion, knee flexion or ankle dorsal flexion angles (F ≤ 1.1, *p* ≥ 0.32, η_p_^2^ ≤ 0.1). Moreover, hip, knee, and ankle dorsal flexion angles decreased through the lift (F ≥ 17.9, *p* ≤ 0.001, η_p_^2^ ≥ 0.64, Figure 2). Post hoc tests showed that dorsal flexion angles decreased just the three first heights for both conditions, whereas hip and knee flexion decreased thorough the whole upward phase at each height (Figure 2).

**Figure 3:**
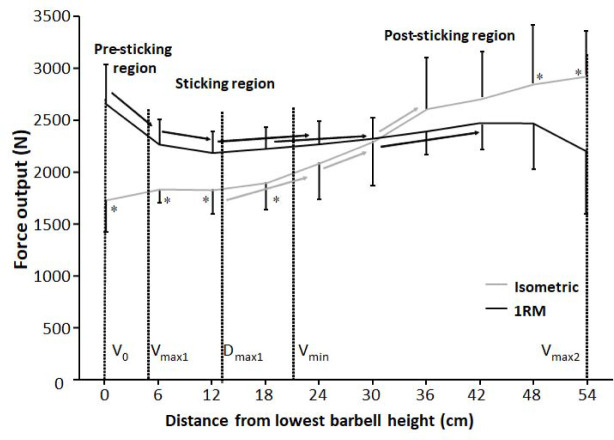
Mean (± SD) vertical force output at the 10 vertical distances from v_0_ for the 1-RM and isometric conditions. * indicates a significant difference in vertical force output between the 1-RM and isometric conditions at this barbell height. → indicates a significant difference between these two barbell heights.

When comparing vertical force output between the 1-RM and isometric squat conditions, no significant effects were observed between the two conditions (F = 1.59, *p* = 0.24, η_p_^2^ ≥ 0.14). Moreover, there was a significant height and condition × barbell height effect (F ≥ 30.46, *p* ≤ 0.001, η_p_^2^ ≥ 0.71). Holm Bonferroni post hoc tests showed that vertical force output was similar at the first 18 cm for the isometric condition before increasing from 12 cm to 24 cm, from 24 cm to 30 cm, and from 30 to 36 cm before becoming stable at the three last barbell heights. For the 1-RM condition, vertical force output decreased from v_0_ the first two heights to 12 cm, before increasing from 12 cm to 24 cm, from 18 cm to 30 cm, and from 30 cm to 42 cm. The different developments of vertical force output resulted in higher vertical force output in the 1-RM at the four first heights, whereas the two last heights had higher force output for the isometric condition (Figure 3).

**Figure 4:**
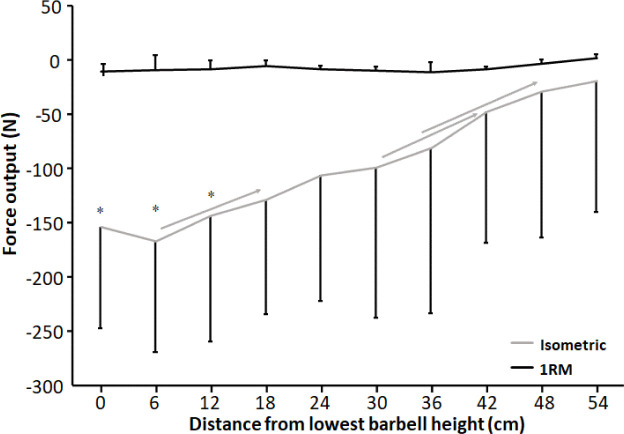
Mean (± SD) anteroposterior force output at the 10 vertical distances from v_0_ for the 1-RM and isometric conditions. Negative value means an anterior force direction. * indicates a significant difference in anteroposterior force output between the 1-RM and isometric conditions at this barbell height. → indicates a significant difference between these two barbell heights for the isometric condition.

For anteroposterior force output, a significant effect of condition, height, and condition * height interaction effect was observed (F ≥ 5.35, *p* ≤ 0.046, η_p_^2^ ≥ 0.37). Holm Bonferroni post hoc tests revealed that anteroposterior force output decreased with increasing height for the isometric condition, while no differences in anteroposterior forces were found between the barbell heights under the 1-RM condition (Figure 4). Also, anteroposterior force output was greater at 0 cm, 6 cm, and 12 cm for the isometric condition compared to the 1-RM condition (Figure 4).

**Figure 5:**
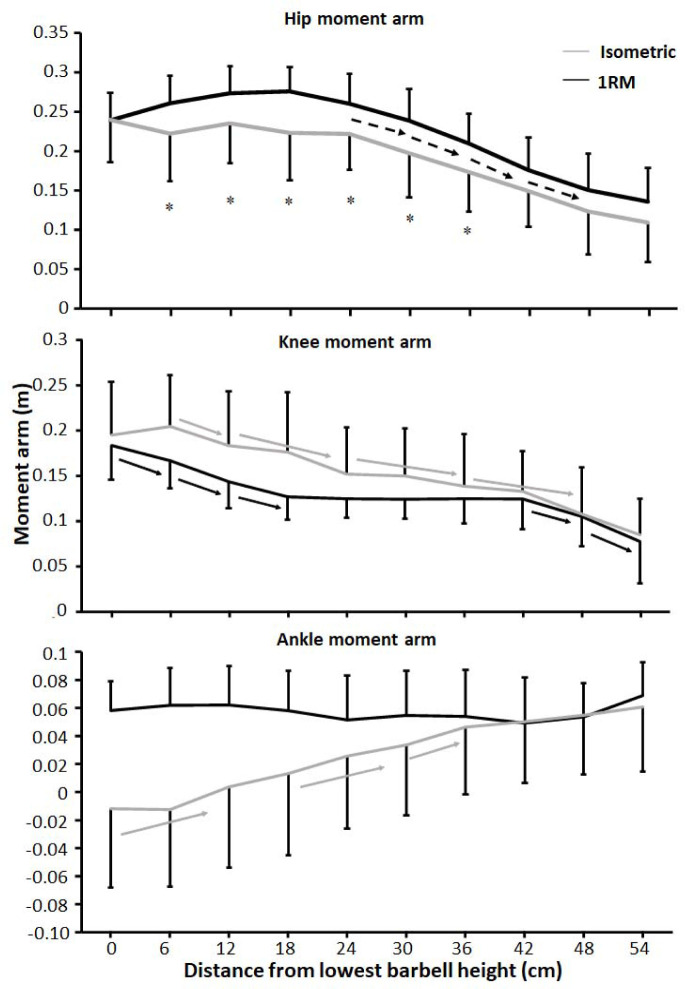
Mean (± SD) hip, knee, and ankle moment arms at the 10 vertical distances from v_0_ for the 1-RM and isometric conditions. * indicates a significant difference between the 1-RM and isometric conditions at this barbell height black → indicates a significant difference between these two barbell heights for the 1-RM squat condition grey → indicates a significant difference between these two barbell heights for isometric squat condition dotted → indicates a significant difference between these two barbell heights for both squat conditions

A significant effect was found for the hip moment arm upon condition and barbell height (F ≥ 10.34, *p* ≤ 0.009, η_p_^2^ ≥ 0.51), whereas the knee extension and ankle plantar flexion moment arms had a significant effect upon barbell height (F ≥ 8.2, *p* ≤ 0.001, η_p_^2^ ≥ 0.45) and condition x barbell height interaction effects (F ≥ 2.3, *p* ≤ 0.022, η_p_^2^ ≥ 0.19). Post hoc tests showed that the hip moment arm was larger for the 1-RM condition compared to the isometric condition. Moreover, the hip moment arm remained stable at the five first barbell heights before decreasing from 30 cm and the rest of the lift (Figure 5). For the isometric condition, the knee moment arm decreased almost linearly through the lift, whereas the knee moment arm decreased the first three barbell heights before remaining stable, resulting in the interaction effect (Figure 5). Post hoc tests revealed that for the isometric condition, the ankle moment arm decreased from v_0_ to 12 cm. Thereafter, the ankle moment arm decreased, before remaining stable at the three last barbell heights for the isometric condition, while for the 1-RM condition, the ankle moment arm remained stable as a dorsiflexion moment arm during the whole lift, resulting in the interaction effect (Figure 5).

**Figure 6:**
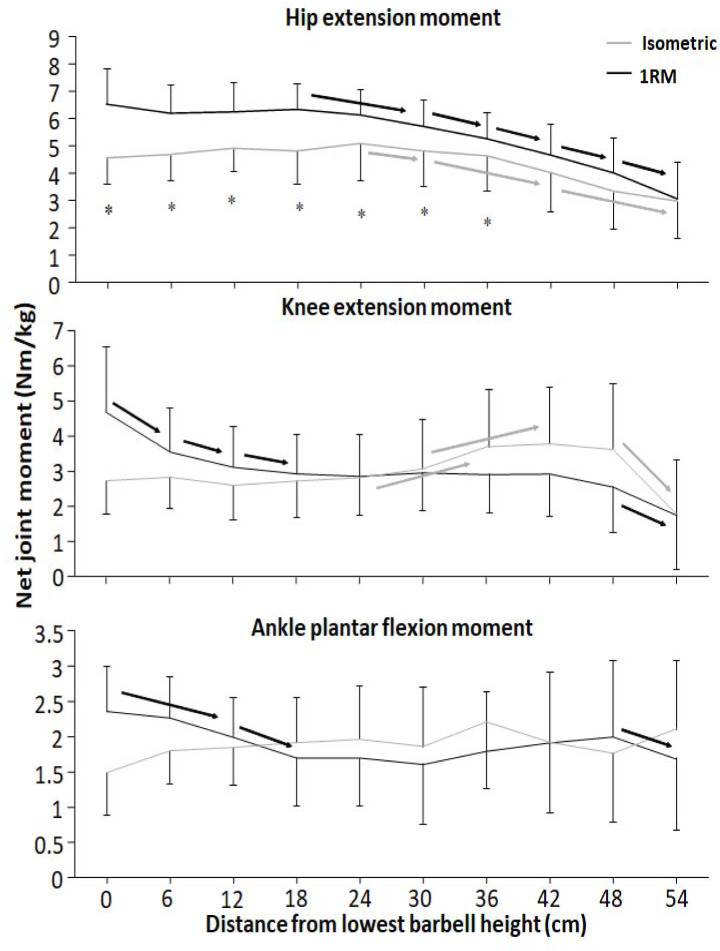
Mean (± SD) net hip extension moment, knee extension moment, and ankle plantar flexion moment at the 10 vertical distances from v_0_ for the 1-RM and isometric conditions. * indicates a significant difference between the 1-RM and isometric conditions at this barbell height black → indicates a significant difference between these two barbell heights for the isometric squat condition grey → indicates a significant difference between these two barbell heights for the 1-RM squat condition

For the hip extension moment, significant effects on condition, barbell height, and condition x barbell height interaction were found (F ≥ 3.7, *p* ≤ 0.03, η_p_^2^ ≥ 0.27). Moreover, significant effects on barbell height and condition x barbell height interaction effect were observed for knee extension moment (F ≥ 7.6, *p* ≤ 0.001, η_p_^2^ ≥ 0.43). Finally, for the ankle plantar flexion moment, a significant condition x barbell height interaction effect was found (F = 4.0, *p* = 0.013, η_p_^2^ = 0.29). Post hoc tests revealed that hip extension moments were greater for the 1-RM condition compared to the isometric condition at the first seven barbell heights (Figure 6). Moreover, the hip extension moment was stable before decreasing from 18 and 24 cm for the 1-RM and isometric conditions. Knee extension moments were stable at the four first barbell heights for the isometric condition before it increased (Figure 5). The 1-RM knee extension moment decreased the three first barbell heights before remaining stable until 48 cm, where the knee extension moment decreased to 54 cm. For ankle plantar flexion moments, the 1-RM condition reduced from v_0_ to 12 cm, from 12 cm to 18 cm, and from 48 cm to 54 cm, whereas the isometric condition had stable plantar flexion moments through the different barbell heights (Figure 6), resulting in larger moment arms for the 1-RM condition the first two barbell heights.

**Figure 7:**
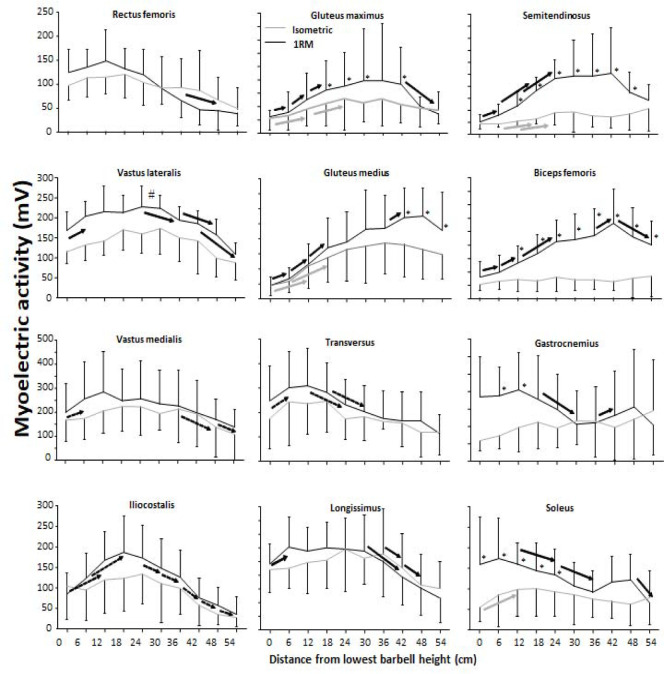
Mean (± SD) sEMG amplitude at the twelve measured muscles at the 10 vertical distances from v_0_ for the 1-RM and isometric conditions. * indicates a significant difference between the 1-RM and isometric conditions at this barbell height. # indicates a significant difference among the squat conditions for all barbell heights. black → indicates a significant difference between these two barbell heights for the 1-RM condition. grey → indicates a significant difference between these two barbell heights for isometric squat condition. dotted → indicates a significant difference between these two barbell heights for both squat conditions.

The EMG profile showed a significant effect for condition on gluteus maximus and medius, semitendinosus, biceps femoris, and vastus lateralis muscles (F ≥ 9.9, *p* ≤ 0.012, η_p_^2^ ≥ 0.52), but not vastus medialis, trapezius pars transversus, erector spinae iliocostalis and longissimus, gastrocnemius, and soleus muscles (F ≤ 3.1, *p* ≥ 0.11, η_p_^2^ ≤ 0.25). Barbell height also had a significant effect on most of the muscles (F ≥ 7.5, *p* ≤ 0.015, η_p_^2^ ≥ 0.45), except the gastrocnemius (F = 0.17, *p* = 0.99, η_p_^2^ = 0.03). Moreover, there was a significant condition x barbell height interaction for all hip extensors and calf muscles (F ≥ 2.7, *p* ≤ 0.01, η_p_^2^ ≥ 0.25). Post hoc comparisons revealed that for most muscles, the sEMG amplitude was higher during 1-RM compared to the isometric condition (Figure 7). For all hip extensors, the sEMG amplitude increased to 24 cm, where it remained stable and decreased again at the last barbell heights. In addition, for vastus muscles, the sEMG amplitude increased to 12 cm barbell height, where activity remained stable to 24 cm (vastus lateralis) and 36 cm before decreasing (Figure 7). Also, erectors muscles together with trapezius pars transversus presented an increased sEMG amplitude during the lower barbell heights before decreasing at the later barbell heights. A greater sEMG amplitude was observed under the 1-RM condition at the lower barbell heights for both calf muscles before decreasing resulting in a similar sEMG profile among the conditions at the higher barbell heights (Figure 7).

## Discussion

The aim of the study was to compare kinetics, kinematics, and the sEMG amplitude of twelve different muscles at ten different positions from v_0_ with 1-RM lifts to investigate the origin of the sticking region in back squats. The main findings were that both conditions had lower force output during the sticking region (Figure 3). Also, all joint moments were highest in the pre-sticking and sticking regions before decreasing during the 1-RM trial (Figure 6), whereas knee extension moments increased again at around 30-cm barbell height for the isometric condition. Furthermore, the hip moment arm reduced first in the post-sticking region, whereas the knee moment arm decreased in the pre-sticking and sticking regions during the 1-RM trial before remaining stable until the last two barbell heights (Figure 5). These findings support our hypothesis that the sticking region occurs due to a poor mechanical position in free-weight back squats. Moreover, force output and the sEMG amplitude were higher under the 1-RM condition compared to the isometric condition for all hip extensor muscles together with the vastus lateralis and soleus muscles (Figure 6), supporting our another hypothesis, that potentiation, and/or better muscle force-velocity and length-tension relationships may occur in 1-RM free-weight back squats due to the descending phase. In addition, the sEMG amplitude increased in the sticking region for vastus muscles, but decreased in the post-sticking region rapidly, whereas the sEMG amplitude for the hip extensors first peaked in the post-sticking region (Figure 7).

Force output under both conditions exposed that vertical force output was lower in the heights representing the sticking region (Figure 3), which is similar to van den Tillaar et al. (2021). Under the 1-RM condition, force output declined from the pre-sticking to the sticking region before increasing again, while force output under the isometric condition was stable to around d_max1_ before increasing at the end of the sticking region and the start of the post-sticking region. In addition, our kinetic data showed stable net hip extension moments until around 18–24 cm in the upward phase where the hip extension moment decreased almost linearly (Figure 6). At the same time, the sEMG amplitude of all measured hip extensors and especially the gluteus maximus peaked after 24 cm (Figure 7), i.e., at the start of the post-sticking region and where the barbell starts to accelerate again (Larsen et al., 2021a). These findings indicate that during the pre-sticking and sticking regions, the hip extension moments are at their highest as shown by the combination of stable maximal force output (Figure 3) and the stable external hip moment arms (Figure 5) during the isometric condition at these different heights. Moreover, at these heights, the hip flexion angle is so large (Figure 2) with a large hip moment arm (Figure 5), that the small internal gluteus maximus and biceps femoris muscle moment arm together with the descending part of the length-tension relationship of these muscles may result in a mechanical disadvantage. Therefore, the muscles capability to contribute to the hip extension moment (Ward et al., 2010) may be reduced. To our knowledge no observations on the length-tension relationship of the gluteus maximus during squats have been done. Nevertheless, the internal moment arms of the gluteus maximus and the hamstring have been observed to decrease with an increased hip flexion angle (Németh and Ohlsén, 1985), potentially reducing their capabilities to contribute to the hip extension net joint moment. Similarly, Ward et al. (2010) showed in a simulation study on squats that the internal moments of the gluteus and the hamstring were lowest at the deepest point of the squat, while the adductor magnus moment was very high at these angles. Thereby, it is speculated that the adductor magnus is the prime mover (Neumann, 2010) to overcome the large hip extension moments during the lower barbell heights, due to its large hip extension moment arm during deep hip flexion (Németh and Ohlsén, 1985).

Furthermore, at the end of the sticking region (18–24 cm in the lift) due to repositioning the knee and the ankle joint, decreasing the moment arm around these joints (Figure 5), we speculate that the gluteus maximus and the biceps femoris increase their internal moments as observed by Ward et al. (2010) and get in a better position to contribute to the hip extension moment. Consequently, they surpass v_min_ (sticking point) in the post-sticking region. Due to the repositioning, the hip moment arm decreases (Figure 5) with increasing height resulting in a decreased hip extension moment during the post-sticking region (Figure 6). It seems that at this point also the gluteus maximus and the biceps femoris get activated more as it was visible by the sEMG amplitude increase during the pre- and sticking regions, reaching its maximum at 18–24 cm in the lift, the start of the post sticking region. Even during the isometric contractions the sEMG amplitudes of the glutes and the semitendinosus (Figure 7) were lower in the pre- and sticking regions, indicating that at these heights the brain does not activate the muscles maximally. We speculate that by information from muscle spindles and Golgi tendon receptors (feed-back loops) in the muscles (e.g., gluteus maximus in the pre- and sticking regions) at a disadvantaged position, the brain would not maximally activate, as this would result in energy loss of the body. However, musculoskeletal stimulation techniques in combination with force output measurements at different joint angles should be performed to confirm this suggestion.

It seems that the body tries to be as effective as possible, since the squat movement starts with knee extension and plantar flexion movements (van den Tillaar, 2015) rather than hip extension as these have lower moment arms (Figure 5), together with peak sEMG amplitudes of the knee extensor and plantar flexor muscles (Figure 7). This results in an increased torso forward lean during the first part of the lift as previously observed (Larsen et al., 2021a, 2021b). However, with large knee flexion angles (pre-sticking and sticking regions) the vastii internal moment arm has been observed to decrease (Kipp et al., 2022), while the net knee extension moment in back squats is stable during these regions (Figure 6). In that way, the vastii muscles may be at a disadvantageous position potentially leading to reduced muscle-specific knee extensor moment production, and thus, the weak link. As Ichinose et al. (1997) suggested, knee extensors may not be capable of producing peak forces until later in the upward phase (around 70 degrees knee flexion, which the knee joint reaches first at the post-sticking region).

A significant interaction effect of force output between the 1-RM and isometric conditions (Figure 3) was observed in which higher forces were found under the 1-RM condition during the pre- and sticking regions, which was likely the result of the processes of utilization of stored elastic energy, and/or the stretch reflex causing potentiation (Walshe et al., 1998), as well as the sEMG amplitude as observed especially in plantar flexors, knee and hip extensor muscles (Figure 7). This was in line with the study of van den Tillaar et al. (2021). In the present study potentiation was also visible by the decrease in force output the first 12 cm and it was lowest at around d_max1_ (Figure 3), which occurred at 0.46 ± 31 s after the start of the upward movement, which is similar to the findings reported by van den Tillaar et al. (2021). This is around the timing that potentiation has been reported to diminish in other studies (van den Tillaar et al., 2012; Walshe et al., 1998). The decrease in force during this part was accompanied by decreases in the ankle and knee moments (Figure 6) and higher sEMG amplitudes of the calf, vastus lateralis muscles for the 1-RM trial compared to the isometric trial before decreasing during the 1-RM condition (Figure 7), meaning that muscle demands are higher. Furthermore, sEMG data from the hip extensor muscles showed a greater sEMG amplitude during the higher barbell heights for the 1-RM condition compared to the isometric condition. This could be due to the larger hip moment arm and extension moment under the 1-RM condition compared to the isometric condition, making the demands harder for the hip extensors (Figures 5 and 6). Another reason could be that due to the dynamic character of the 1-RM condition, the different muscles are at other parts of the length-tension and force-velocity relationships of the muscles. Thereby, these muscles are in a more efficient part of these relationships to be activated via different feedback loops by the brain.

Moreover, force output increased more rapidly from 30–36 cm (post-sticking region) for the isometric condition compared to the 1-RM condition. This was probably achieved with the increased knee extension moment at around 30–36 cm barbell height for the isometric condition (Figure 6). The knee extension moment peaked at around 70**°** degrees knee flexion for the isometric condition, which is at the same angle that has been reported to be the optimal vastus lateralis fascicle length to produce force (Ichinose et al., 1997).

The barbell kinematics in our study were comparable with previous studies investigating the sticking region in back squats (Larsen et al., 2021a, 2021b; Saeterbakken et al., 2016; van den Tillaar, 2015, 2019; van den Tillaar et al., 2014) indicating that their true 1-RM was measured. However, barbell heights for the sticking region (from 5 to 21 cm) were longer than in the study by van den Tillaar et al. (2021) (5 to 10 cm). This difference probably occurred because they used the Smith machine for the 1-RM condition, thereby reducing the horizontal movements and in that way leading to a shorter sticking region.

The present study has some limitations. The major limitation is that most of the speculated characteristics for why the sticking region occurs were not measured (i.e., muscle length and internal moment arms), thus we referred to interpretations from previous studies, and not variables actually measured in this study to explain why the sticking region occurs in back squats. Therefore, our interpretations should be taken with caution, since our study design did not allow to establish causation. Moreover, we observed anteroposterior forces under the isometric condition. This resulted in smaller external hip and ankle moment arms for the isometric compared to the 1-RM condition. This is because under the isometric condition, participants produced the anterior forces against the ground, which resulted in force travelling backwards in the posterior direction. This is a significant limitation of our study which may have contributed to potential differences in the observed kinetics and therefore the kinematics and the sEMG amplitude. Notably, future studies should use musculoskeletal modelling techniques to quantify muscle forces and their respective internal moment arms, and thus their contributions to the hip extensor, knee extensor, and ankle plantar flexion moments.

## Conclusions

Based on our results, we propose that the sticking region occurs because of reduced force output in the pre-sticking and sticking regions in back squats among resistance-trained males. The reduced force output is probably a combination of suboptimal internal moment arms, length-tension relationships of the gluteus maximus, hamstring and vastii muscles in the pre-sticking and sticking regions to overcome the large extensor moments together with diminishing potentiation from the pre-sticking to the sticking region.

Thereby, as a practical implication when lifting maximal loads, encountering the sticking region in back squats is likely inevitable due to suboptimal gluteus maximus and vastii moment arms and diminishing potentiation. However, for individuals aiming to enhance their squat 1-RM, prioritizing the development of maximal strength of the gluteus maximus, hamstrings and vastii muscles seems crucial to increase 1-RM and overcome the sticking region with higher barbell loads.
